# Tactics for Drawing Youth to Vaping: Content Analysis of Electronic Cigarette Advertisements

**DOI:** 10.2196/18943

**Published:** 2020-08-14

**Authors:** Laura L Struik, Sarah Dow-Fleisner, Michelle Belliveau, Desiree Thompson, Robert Janke

**Affiliations:** 1 School of Nursing Department of Health and Social Development University of British Columbia Okanagan Kelowna, BC Canada; 2 School of Social Work Department of Health and Social Development University of British Columbia Okanagan Kelowna, BC Canada; 3 Library University of British Columbia Okanagan Kelowna, BC Canada

**Keywords:** qualitative research, electronic nicotine delivery systems, marketing, advertisement, youth, vaping

## Abstract

**Background:**

The use of electronic cigarettes (e-cigarettes), also known as vaping, has risen exponentially among North American youth in recent years and has become a critical public health concern. The marketing strategies used by e-cigarette companies have been associated with the uptick in use among youth, with video advertisements on television and other electronic platforms being the most pervasive strategy. It is unknown how these advertisements may be tapping into youth needs and preferences.

**Objective:**

The aim of this 2-phase study was to examine the marketing strategies that underpin e-cigarette advertisements, specifically in the context of television.

**Methods:**

In phase 1, a scoping review was conducted to identify various influences on e-cigarette uptake among youth. Results of this scoping review informed the development of a coding framework. In phase 2, this framework was used to analyze the content of e-cigarette advertisements as seen on 2 popular television channels (Discovery and AMC).

**Results:**

In phase 1, a total of 20 articles met the inclusion criteria. The resultant framework consisted of 16 key influences on e-cigarette uptake among youth, which were categorized under 4 headings: personal, relational, environmental, and product-related. In phase 2, 38 e-cigarette advertisements were collected from iSpot.tv and represented 11 popular e-cigarette brands. All of the advertisements tapped into the cited influences of youth e-cigarette uptake, with the most commonly cited influences (product and relational) tapping into the most, at 97% (37/38) and 53% (20/38), respectively.

**Conclusions:**

The findings highlight the multidimensional influences on youth uptake of e-cigarettes, which has important implications for developing effective antivaping messages, and assist public health professionals in providing more comprehensive prevention and cessation support as it relates to e-cigarette use. The findings also bring forward tangible strategies employed by e-cigarette companies to recruit youth into vaping. Understanding this is vital to the development of cohesive strategies that combat these provaping messages.

## Introduction

The use of electronic cigarettes (e-cigarettes), also known as vaping, among youth is a pressing public health concern in the United States and Canada. Importantly, the initiation and use of vaping products among youth have been associated with immediate and lasting health consequences [1-3]. Yet, according to a recent report, nearly 1 in 3 high school–aged students and 1 in 7 middle school–aged students report vaping [4,5], which reflects a significant increase in use. Specifically, between 2017 and 2019, vaping among youth in the United States has more than doubled, with reported use increasing from 4% to 9% among 8th graders, 8% to 20% among 10th graders, and 11% to 25% among 12th graders [5]. Canada reflects similar trends, with past 30-day use of e-cigarettes up from 10% in 2016-2017 to 20% in 2018-2019 among youth in grades 7 to 12 [4].

While e-cigarettes entered the North American market as a cessation tool in 2008, e-cigarette use among non-smoking youth suggests that cessation is not a primary reason for use in this age demographic. According to recent statistics, only 3% of Canadian youth in grades 7 to 12 are current smokers, and 20% are current e-cigarettes users, suggesting that upwards of 17% of e-cigarette users were originally non-smokers [4]. In addition, among youth who do smoke combustible cigarettes, fewer than 8% report using e-cigarettes for smoking cessation [6]. In relation to smoking cessation, emerging evidence is inconclusive as to the effectiveness of e-cigarettes as a cessation method for youth [7], and some research even suggests that it contributes to ongoing nicotine addiction [8]. Even more concerning is that vaping among youth has been linked to a 3 times greater likelihood to try traditional cigarettes [9-12]. These findings have contributed to what the US Department of Health and Human Services Secretary Alex Azar described as “an epidemic of youth e-cigarette use, which threatens to engulf a new generation in nicotine addiction” [13].

Marketing strategies used by e-cigarette companies have been associated with the uptick in vaping among youth [10]. Many studies have found that the promotion of e-cigarettes through various channels (eg, television [TV], social media influencers) has lent to increased positive perceptions of vaping and intentions to use vaping products and contributed to e-cigarette uptake among youth [14-20]. The use of TV as a mode of marketing has been the most recent focus for e-cigarette companies. Between 2018 and 2019, JUUL, the most popular e-cigarette brand in North America [21,22], spent US $57 million on TV advertisements to promote their products [23]. While JUUL claims that these advertisements are aimed at helping adults find a healthier alternative to smoking, many public health advocates are concerned these advertisements may also attract youth [24], and research confirms that these advertisements increase exposure to their products, which subsequently increases the likelihood of use by youth [25,26]. For example, in a recent study, it was found that exposure to e-cigarette advertisements among youth is not uncommon, with 28% of youth in the United States, 17% of youth in Canada, and 21% of youth in England having seen a vaping advertisement [25]. Furthermore, nearly 40% of youth across all three countries reported that the advertisements made vaping look appealing, and about 44% of youth perceived that vaping advertisements targeted non-smokers [25]. Indeed, skepticism is warranted in relation to how e-cigarettes are portrayed in advertisements.

Subject to the Food and Drug Administration Family and Tobacco Control Act in the United States [27] and the Tobacco and Vaping Products Act in Canada [28], e-cigarette companies are not permitted to market or sell their products to youth. While this broad stipulation is helpful, we know little about what marketing to youth truly looks like in order to deem advertisements to be appropriately adhering to this provision. Given that advertisements clearly influence uptake among youth, it is important that the ways in which the advertisements are tapping into youth needs and preferences be examined. Thus, the aim of this study was two-fold: first, to conduct a scoping review to identify the known influences associated with youth vaping and second, to analyze the content of e-cigarette advertisements using content analysis to identify and describe overlapping themes.

## Methods

### Aim 1: Scoping Review of Vaping Influences on Youth

A scoping review of the literature was conducted to identify various empirically supported influences on e-cigarette uptake among youth, which was then used to develop the coding framework to analyze influences present in vaping ads. The search strategy was developed by the research team, including a research librarian and experts in youth vaping and substance use, and based on a methodology reported by Kinouani and colleagues [29]. The scoping review used two databases, Medline on the OVID platform and CINAHL on the EBSCO platform, using a combination of keywords and database-specific subject headings. The search strategy from Medline is included in Multimedia Appendix 1. Inclusion criteria were studies that were in North America, published in English, focused on correlates of and influences on e-cigarette use, and focused on youth. Also, both qualitative and quantitative methodologies and literature reviews were included. Exclusion criteria were studies that were a surveillance of e-cigarette use, focused on traditional cigarette use, biomedical studies, or not focused on youth.

The initial search, limited to articles in English with an abstract available, resulted in 937 articles across both databases. Results were uploaded to RefWorks, a bibliographic management software package, and removal of duplicates resulted in 855 articles left for screening. Using the exclusion and inclusion criteria, two research assistants screened the article titles and abstracts independently, with disagreements resolved in collaboration with the first and second authors. After abstract and title screening, the research team members screened 37 articles at the full-text level, which resulted in 20 final articles for analysis. For the final 20 articles, two research assistants extracted all significant influences on youth vaping. Using Excel, the cited influences on youth e-cigarette uptake were listed. Using conventional content analysis, categories and subcategories were inductively derived [30]. Two research team members extracted data from the first 5 articles, generating initial codes and categories for the different influences on youth uptake of e-cigarettes. The whole research team met to achieve consensus on the approach to data extraction. The team then generated an overall framework of categories and subcategories. Two research team members then extracted data about the influences on vaping among youth from the remaining articles. Once all the articles were reviewed and data were extracted, the team met again and refined the coding framework into 4 overall categories associated with youth vaping influences.

### Aim 2: Application of Coding Framework to e-Cigarette Advertisements

#### Data Collection of e-Cigarette Advertisements

A total of 38 e-cigarette advertisements were collected using the freely available iSpot.tv, which is a real-time television advertisement platform [31]. Using the iSpot.tv platform, we searched for industry-driven advertisements related to e-cigarettes that were aired in North America between December 15, 2019 and January 15, 2020. Using this platform ensured that the advertisements collected for this study were generated by e-cigarette companies versus informal or individually developed e-cigarette promotions, like those posted on social media sites. Initially, common e-cigarette brands were searched including BLU, JUUL, FIN, LOGIC, and VUSE, which resulted in 29 nationally aired advertisements. The terms “vape,” “vaping,” and “e-cigarettes” were also used to search for advertisements, which resulted in 8 additional advertisements for the brands CUE, FreeBoxMod, O2PUR, VCHIC, and VERO. The search for advertisements was completed between December 15, 2019 and January 15, 2020.

#### Advertisement Content Analysis

The coding framework developed from the scoping review of influences on youth vaping was used to deductively analyze the content of the advertisements. Deductive content analysis is the process of applying data to a pre-existing framework [30]. First, two researchers coded the same 2 advertisements using the a priori themes developed through the systematic review of the literature on vaping influence for youths. The research team then met to discuss the application of the coding framework and reached consensus regarding the coding process. The two researchers then finished applying the framework to the advertisements. In addition to the framework, we coded the look and feel of the ads to capture demographic and other contextual data (eg, age, race, and sex of individuals in the ads and location setting of the ads). Finally, we noted key messages in the narratives and taglines for each advertisement.

## Results

### Scoping Review Description

The scoping review generated a coding framework that revealed 4 overall categories associated with youth vaping, including personal, relational, environmental, and product-related influences, with 16 subcategories. Personal influences were related to whether youth reported vaping to remove negative affect (eg, daily stress, anxiety, boredom), recreation was related to youth vaping for the purposes of having fun (eg, doing tricks), and finally curiosity was related to vaping to experiment with something new. Relational influences were related to family approval, such as getting positive message about vaping from parents or siblings, and included whether parents, siblings, or peers were using e-cigarettes. This category also included whether youth viewed vaping as enhancing social capital (eg, viewing vaping as a necessary part of social events) and enhancing social acceptance (eg, using e-cigarettes to fit in with peers). Environmental factors were related to external factors influencing ease of access or use (eg, easy to use and obtain e-cigarettes) and the impact of cost related to vaping. Finally, we found that elements related to vaping products were key influencers for youth. These included the ability to use the product discreetly (eg, no bad smell), have a positive sensory experience (eg, good flavors and better “buzz” than cigarettes), and use a new or innovative product. Lastly, perceptions that vaping was less harmful than cigarettes and that it could be used to support smoking cessation influenced e-cigarette uptake among youth.

Of the 20 articles reviewed, there were 57 different cited examples of product-related influences, 41 relational influences, 19 personal influences, and 11 environmental influences. Of note, an article could have more than one example of an influence type. For example, Ickes and colleagues [32] reported ease of use or access, peer use, good flavors, and low cost as some influences of current use and initial use. Among subcategories, perceptions of vaping being less harmful than cigarettes and peer use were among the most cited influences for youth e-cigarette use, followed closely by the perception of vaping as a positive sensory experience and use as a smoking cessation aid. For instance, 81% of youth reported initiation of e-cigarette use because a friend vaped, and 80% reported continued e-cigarette use because of the good flavors. See Table 1 for final categories and subcategories with citations and examples and Multimedia Appendix 2 for references of articles from the scoping review.

**Table 1:** Influences on youth electronic cigarette (e-cigarette) uptake. See Multimedia Appendix 2 for the reference list.

Influences		Articles	Example
**Personal**			
	Removal of negative affect	[5,7,9,11,18]	Participant reports: “I have issues with anxiety…sometimes if I’m dealing with sensory overload…[vaping] really helps” [5]
	Recreation	[2,5,9,11,18]	22.4% reported vaping to have a good time, 21.6% to relax, and 23.5% to reduce boredom [18]
	Curiosity	[2,8,9,11-14,17,18]	95% of youth reported curiosity as the reason for initiating vaping [11]
**Relational**			
	Family approval	[2,6,12,13,17,19,20]	6.8 times greater risk of vaping if there is an e-cigarette user at home [6]
	Parent use	[1,4,8,10]	Higher rate of youth vaping (14%) associated with maternal e-cigarette use [10]
	Sibling use	[2,10,17]	Participant reports: “I got it [e-cigarette] from my older brother; he was with his friends…he told me I should try it” [2]
	Peer use	[2,4-6,8,10,12-15,17,19,20]	Friend vaping associated with an increased frequency of use (*r*=.30, *P*<.001) [20]
	Enhance social capital	[5,7,13,17,18]	Participant reports: “[Vaping] tasted good and it was mostly a social thing. It looked cool, and I wanted other people to think that I looked cool” [5]
	Enhance social acceptance	[1,5,6,8,10,14,15,17,20]	28% of youth who ever vaped and 46% with current use reported vaping to feel more comfortable in social situations [7]
**Environmental**			
	Easy to access or use	[2,4,10,11,13,16,19,20]	91% of youth reported “ease of use” as their reason for continued use of e-cigarettes [11]
	Cost	[8,13,19]	2.5%-3.9% reported vaping because they cost less than cigarettes [19]
**Product**			
	Discreet	[8-13,18,19]	1.76 times more likely to try vaping because it can be hidden from adults [13]
	Positive sensory experience	[2,5,8-14,17-20]	42% youth reported ‘good flavors’ as a reason for first use [8]
	Less harmful	[2-8,10,11,13,15-17,19]	52-54% youth with past 30-day use reported vaping was not harmful to their health [7]
	New or novel product	[2,12-15,17,18,20]	72% reported trying e-cigarettes because they were something new, cool, or fun [12]
	Smoking cessation	[4,5,8-13,15,17-19]	8.5% report using e-cigarettes to quit smoking [9]

### Advertisement Descriptions and Context

Among the 38 e-cigarette advertisements reviewed, 11 advertisements were for BLU, 7 for JUUL, 6 for VUSE, 1 for CUE, 3 for FIN, 3 for LOGIC, 3 for O2PUR, 2 for VERO, 1 for FreeBoxMod, and 1 for VCHIC. Of the 38 advertisements reviewed, 73.7% (28/38) included people, with 16 of 28 advertisements featuring individuals as couples or in groups. Most advertisements featured people who appeared to be white (25/38) or black (10/38), with 9 advertisements showing more than one race in the advertisement. Among the 28 advertisement showing people, 17 included both male and female actors, 6 had male actors only, and 5 had female actors only. Additionally, among advertisements featuring people, most showed individuals who appeared to be 19-30 years old (15/28) or 31-40 years old (14/28). The most common settings for advertisements were during recreational activities (eg, party or camping; 13/38), in a city (11/38), or within an individual’s home (10/38), followed by advertisements showing a person’s workplace (5/38). Many advertisements showed individuals using e-cigarettes in a variety of settings. For example, advertisements showed individuals using e-cigarettes at home, work, school, in social settings (eg, at a bar), and while doing recreational activities (eg, biking, camping). Many of the advertisements (eg, VUSE) used incredible graphic designs with the use of vibrant colors, animation, music, and setting transitions. While all the advertisements utilized action-oriented videography to market their products, they varied in terms of being virtual animation only, real-life settings, or a mix of both. Finally, advertisements also frequently included narration and taglines related to the product. Phrases included, “Take back your freedom” (BLU), “Make the switch” (JUUL), “Satisfying; It’s that simple” (LOGIC), and “Real draw, real taste, real satisfaction” (VUSE).

### Advertisements and Vaping Influences

Among the 38 advertisements reviewed, all 4 of the main influences identified in the scoping review (personal, relational, environmental, and product) and the majority of subthemes in the framework were present. Parental and sibling use and exposure to advertisements were not present in the advertisements analyzed. However, there was mention of siblings and spouses being more accepting of vaping than smoking (eg, JUUL). The majority of advertisements (97%; 37/38) had at least one influence related to the product, and 20 advertisements (20/38, 53%) included at least one element related to relational influences. Additionally, 16 advertisements (16/38, 42%) included elements related to personal influences, and 9 (9/38, 24%) included environmental influences. The most common influences present in vaping advertisements included the product as new or innovative (30/38), the positive sensory experience of vaping (20/38), the ability of vaping to enhance social acceptance (18/38), and vaping as an alternative to smoking (18/38). In sum, all the advertisements included at least one influence, with an average of 4.39 (SD 0.31) influences per advertisement (range 1-10).

Furthermore, advertisements often emphasized the ability to derive “satisfaction” from the product (eg, LOGIC, VUSE, CUE, and FIN). Common words and phrases included “100% flavor,” “satisfaction at last,” “satisfaction,” “real satisfaction,” “unrivaled taste satisfaction,” and “a truly satisfying taste” and encouraged consumers to “draw” and “taste” the array of flavors. Additionally, companies like BLU, JUUL, VERO, and VCHIC presented their product as an “alternative” to smoking and included phrases like “make the switch” and “rise from the ashes.” See Table 2 for the number of advertisements with a particular influence and examples of how the influence was presented within the advertisement.

**Table 2:** Content analysis of vaping advertisements.

Influences		Number of ads	Examples
**Personal (n=16)**			
	Removal of negative affect	4	“Now that I’ve switched to BLU, I feel so much better about myself” (BLU 1056056)
	Recreation	4	Actor (man) hiking, bicycling, and intent to race car while vaping (BLU 1075930)
	Curiosity	10	“Try CUEThese risk free, and change your life” (CUE 1717816)
**Relational (n=20)**			
	Family approval	4	Adult son on the switch from smoking to vaping based on his mother’s suggestion. “This [the switch to JUUL] came from her [Mother], really.” (JUUL 2059571)
	Parent use	0	No explicit mention of parent use leading to vaping.
	Sibling use	0	No explicit mention of sibling use leading to vaping.
	Peer use	3	“It was a friend of mine that said, why wouldn’t you just try the JUUL?” (JUUL, 2060997)
	Enhance social capital	12	“I can whip out my BLU and not worry about scaring that special someone away,” (BLU 1056056).
	Enhance social acceptance	18	“There was a time when no one was offended by it [smoking]. That time has come again” (FIN, 1044824)
**Environmental (n=14)**			
	Easy to access or use	9	“Truly vaping made easy.” (CUE 171816)
	Cost	13	“VCHIC saves me over $150 dollars a month” (VCHIC 1089981)
**Product (n=37)**			
	Discreet	17	“A better way to enjoy everything you love about smoking, only without the smell” (CUE 1717816)
	Positive sensory experience	20	“Real draw, real taste, real satisfaction” (VUSE, 2148838) “Four new flavors to awaken your senses (VUSE, 1266750)
	Less harmful	5	“Definitely has all the stuff I want, and not all the bad stuff” (FIN 1148496)
	New or novel product	30	“Innovation has changed the world, moving us all forward, isn’t it time smoking changed too?” (VUSE 2148838)
	Smoking cessation	18	“I was a pack -a-day smoker for more than 30 years….(until I switched to JUUL),” (JUUL, 2346024)

## Discussion

### Principal Findings

The factors influencing youth vaping are varied, complex, and multidimensional. A scoping review of the extant literature identified 16 major influences associated with youth vaping, which fell under the categories of personal, relational, environmental, and product-related factors. These findings indicate that vaping among youth is prompted by a variety of factors and not just one factor alone. To date, however, prevention campaigns have typically focused on addressing perceptions of harmlessness. For example, a recent review of 21 prevention interventions across North America confirmed that these efforts frequently appeared to be designed to teach youth about the dangers of vaping and encouraging them to refrain from or to stop vaping [33]. While these efforts are a much-needed step forward, the findings from this scoping review present more than just perceptions of harmlessness as influencing youth uptake and include curiosity, social factors, and stress and anxiety, to name a few. The provision of information on the harms is a common response to substance use [34], but with little evidence to support its effectiveness [35,36]. While it is important to relay information and implement protective policies, it is also important to acknowledge other factors at play. Hyshka [34] suggested that prevention interventions for substance use target the social determinants rather than the individual behavior to improve young people’s health and well-being. In this regard, prevention efforts for vaping would benefit from a deeper understanding of the various and relevant reasons and pathways to vaping among youth populations and should develop holistic interventions driven by youth.

The TV advertisements reviewed were found to tap into almost all of the reasons that youth cite for taking up e-cigarettes. The most highly cited reasons were most prominent in the ads, including a focus on relational aspects of vaping and product-related benefits, such as a positive sensory experience. Similar to the findings of the present study, a recently published focus group study that analyzed e-cigarette advertisements with 39 non-vaping adolescents found that the perceived social benefits, like increased friendships, and product-related appeals, like the innovative design and variety of flavors, presented in the advertisements were major draws to trying e-cigarettes [37]. The present study builds on these findings even further and adds to a growing evidence base that adolescents are indeed the target market for e-cigarette advertisers, despite claims that this is not the case. In addition, findings from this study shed light on how advertisements are successfully drawing this demographic into vaping, offering tangible results that tobacco control advocates can draw upon to inform the development of policies and interventions to combat these marketing strategies.

Rather than emphasizing the use of e-cigarettes to address nicotine addiction, the advertisements emphasized e-cigarettes as a solution to maintain nicotine dependence by portraying e-cigarettes as an innovative way to get the nicotine fix most commonly associated with cigarettes. While phrases like “make the switch” by JUUL may appear to suggest e-cigarettes as tools for smoking cessation, it does not appear to be the intention of these advertisements or the e-cigarette companies. For example, JUUL advertisements (eg, JUUL, 2060997) include a warning that says: “JUUL is not a smoking-cessation product and has not been approved by the FDA for the treatment, prevention, or cure of any specific disease or condition.” Despite this, e-cigarette companies like JUUL still benefit from lax regulations and the ability to advertise their products as an alternative nicotine product. Adding to this, many of the advertisements ridiculed traditional cigarettes for being outdated. In this regard, e-cigarette companies are differentiating themselves from traditional cigarettes by minimizing, and even attempting to eradicate, any relationship with the stigmatized cigarette. To promote e-cigarettes as a solution to get a nicotine fix and, at the same time, purposefully convey e-cigarettes as unrelated to cigarettes are in direct opposition to the mandate that they are marketed as a cessation aid for cigarette smokers. Additionally, most e-cigarette pods contain up to three times the amount of nicotine compared to a pack of combustible cigarettes [38], which further contradicts the notion that e-cigarettes are for the purposes of cessation.

### Comparison to Prior Work

That the TV advertisements largely tapped into the influence of peers and relationships, one of the most commonly cited influences on uptake in the scoping review results, is noteworthy. Peers become an important influence during the adolescent stage of development, with social networks and social acceptance essential parts of positive identity development [39-41]. This establishment of identity and acceptance in a social group has been termed entitativity [42,43]. A high sense of entitativity is associated with improved peer relationships and self-efficacy, and a low sense of entitativity is associated with poor views of self and peers [42]. Entitativity occurs with youth as they identify with a social group to gain social acceptance and respect [41]. For youth, entitativity alters perceptions and beliefs and increases willingness to participate in risky health-related activities [41]. For example, entitativity in smoking groups has been found to lend to more homogenous opinions and reinforces positive perceptions of smoking [43,44]. Furthermore, individuals that strongly identify with smoking groups are more likely to resist antismoking campaigns [44]. Similar to smoking, e-cigarettes allow youth to form a community based on the inclusion criteria of vaping, which enables opportunities for increasing social networks and establishing a group identity [45,46]. Narratives of social identity and belonging around e-cigarette use have a powerful impact on youth, not only for shaping positive attitudes around vaping but also for resisting prevention efforts. Indeed, the advertisements reviewed in the present study may serve as a pre-emptive measure to keep youth vaping despite the roll out of anticipated antivaping campaigns and interventions.

Despite most of the advertisements being 30-60 seconds long, they still managed to convey e-cigarettes as less harmful than traditional cigarettes by presenting them as the cleaner alternative to cigarettes or as a cessation aid. This is similar to the findings of Richardson and colleagues [47], who found that online advertisements prioritized messages of reduced harm within their short window of advertising time. This is concerning because there is no basis to make claims of reduced harm, and a lack of knowledge should not be conflated with no harm. Even more concerning is that youth are particularly vulnerable to these messages [24,48]. Making unfounded claims around harmlessness supports misperceptions of e-cigarette safety, promotes uptake, delays cessation, and encourages dual use of tobacco products [49,50], the latter of which has recently been found to significantly compound long-term health risks, including chronic obstructive pulmonary disease [51] and stroke [52]. Making modified risk claims, therefore, contributes significantly to the burden of population-level harm.

It is also noteworthy that the advertisements promoted the ability to use e-cigarettes in a variety of contexts, including contexts where traditional cigarette smoking is banned, such as indoors or in urban areas, because of their sleek design and lack of odor [53,54]. Vaping is essentially a way to avoid smoke-free policies due to a current lack of regulations [55,56]. In this regard, these advertisements reinforce already problematic patterns of use among youth, such as hiding it from parents [54,57] or using e-cigarettes in challenging contexts, like schools [58,59]. By reinforcing the ability to vape anywhere, harmful patterns of use will likely increase.

A prominent theme in the ads was the incitement of curiosity through presentation of vaping as something new. Curiosity is a predictor of youth starting to vape, and curiosity about vaping is often associated with increased exposure to advertisements [60-62]. e-Cigarette use in the advertisements is framed in such a way as to peak curiosity through increasing novelty, going against the norm, and messages that these products are innovative. Curiosity is a powerful experience for youth that appears to inform both smoking and vaping behavior. These findings add further support to the idea that exposure to advertisements is informing youth vaping behavior.

Using stunning sensory appeals, such as through graphical displays that emphasize the visual appeal of sleek technological designs, and the pleasurable taste and smell of e-cigarettes, the advertisements play to the sensation-seeking needs of youth. Adolescence is a period of psychosocial development where it is more likely that an individual will engage in sensation seeking than at other developmental stage [63]. Other researchers confirm that e-cigarette advertisements capitalize on this sensation-seeking behavior by emphasizing pleasurable sensations, such as flavors or odor produced by the e-juice [37,64-66]. The advertisements reinforce the belief that e-cigarettes will satisfy the sensation-seeking needs of youth by highlighting the sensory experience that comes with e-cigarette use in their animations, graphics, and music. The high rates of vaping among youth can be attributed to these strategic, developmentally targeted, marketing tactics.

### Future Research

Youth vaping is a concern, and there is a growing need for comprehensive strategic plans to curtail youth vaping. One strategy is to provide stronger and clearer regulations for vaping ads. Given the findings of this study, we see that many advertisements inadvertently or directly included elements in advertisements that tap into key influences of vaping among youth. Thus, we can conclude that the current regulations are not having the intended impact. Findings from this study suggest that future research should explore the development and impact of regulations that consider the personal, relational, environmental, and product influences that are most meaningful for youth and ensure that these are limited in vaping advertisements. For example, regulations should restrict the sensory-appeal tactics in advertisements.

In addition, while this study looked at influences on e-cigarette use among the general population of youth and how advertisements tapped into these influences, it would be interesting to examine differences according to developmental stage. Broadly, adolescence is a time when youth are learning to assert their independence, try new tasks, and seek out support from peers more so than parents, which influences risky health behaviors like vaping [67]. Thus, future research should look at stages of adolescent development in relation to susceptibility to advertising and motivations around vaping (eg, early adolescence [10-13 years old], middle adolescence [13-15 years old], late adolescence [15-19 years old]). As seen in other tobacco prevention efforts [68], this would lend to more targeted efforts to curb youth vaping.

Research aimed at developing and evaluating interventions to curtail youth vaping is also needed. The findings of this study provide preliminary evidence for influencing factors that may be taken into consideration in interventions. Further research is needed to explore and understand how different populations of youth perceive e-cigarette advertisements and how the advertisements appeal to them. Finally, research that examines how different aspects of the advertisements may be playing to vulnerable populations, such as low-income groups, needs to be explored further.

### Limitations

The findings of this study are limited to North American applications. Factors that influence use and the tactics of e-cigarette advertisements in other countries may be different. In addition, we do not know if reasons for uptake of e-cigarettes are the same for adults and is an area for future research. It is also important to note that the advertisement selection was limited to the iSpot.tv platform, and there may be other ads with nuances not captured in this study. Furthermore, while not all e-cigarette companies have TV advertisements, it is important to note that this study represents 10 out of over 450 brands now available [69]. Finally, the advertising landscape for e-cigarettes is a rapidly evolving space, in terms of content and delivery channels, and it is important to consider the study findings in light of changes in the field.

### Conclusions

The findings of this study reveal the multidimensional influences on youth uptake of e-cigarettes. Importantly, this study also reveals how these influences are leveraged in e-cigarette advertisements. This contributes to a more nuanced understanding of how e-cigarette companies market their products and to whom. Understanding this is vital to our understanding of how to combat them.

## Appendix

Multimedia Appendix 1 Medline search strategy.

Multimedia Appendix 2 Scoping review references.

## Abbreviations

e-Cigarettes: electronic cigarettes
TV: television
