# Supplementary material for: Tactics for Drawing Youth to Vaping: Content Analysis of Electronic Cigarette Advertisements
Source: J Med Internet Res. 2020 Aug 14;22(8):e18943. doi: 10.2196/18943 (PMC7455879; doi:10.2196/18943)
Supplement: Multimedia Appendix 1 [file jmir_v22i8e18943_app1.docx]

**Appendix 1. Medline Search Strategy**

| **Database:**  Ovid MEDLINE(R) and In-Process & Other Non-Indexed Citations <1946 to October 28, 2019> | | |
| --- | --- | --- |
|  | **Search Terms** | **Results** |
| 1 | (child* or youth* or "young adult*" or teenager* or kid* or "school aged" or adolescent* or "middle school*" or "high school*").tw. | 1999685 |
| 2 | exp Child/ | 1880112 |
| 3 | exp Adolescent/ | 1993255 |
| 4 | or/ 1-3 | 3852318 |
| 5 | (reason* or factor* or motivat* or decision or decide).tw. | 3853033 |
| 6 | exp Motivation/ | 166576 |
| 7 | exp Decision Making/ | 197101 |
| 8 | or/ 5-7 | 4081705 |
| 9 | ("Electronic Nicotine Delivery Systems" or "e cigarettes, e-cigarettes" or "electronic cigarettes" or "vaping" or "JUUL" or "e-cigs" or "vape pens").tw. | 2639 |
| 10 | exp Vaping/ or exp Electronic Nicotine Delivery Systems/ | 3151 |
| 11 | or/ 9-10 | 4288 |
| 12 | 4 and 8 and 11 | 438 |
| 13 | limit 12 to (abstracts and English language) | 429 |
