# Supplementary material for: Tactics for Drawing Youth to Vaping: Content Analysis of Electronic Cigarette Advertisements
Source: J Med Internet Res. 2020 Aug 14;22(8):e18943. doi: 10.2196/18943 (PMC7455879; doi:10.2196/18943)
Supplement: Multimedia Appendix 2 [file jmir_v22i8e18943_app2.docx]

**Appendix B. Scoping review references**

1. Alcala HE, Albert SL, Ortega, AN. E-cigarette use and disparities by race, citizenship status and language among adolescents. *Addictive Behaviors*. 2016; 57: 30-34. doi:10.1016/j.addbeh.2016.01.014
2. Alexander JP, Williams P, Lee YO. Youth who use e-cigarettes regularly: A qualitative study of behavior, attitudes, and familial norms. *Preventive Medicine Reports.* 2018; 13: 93-97. doi:10.1016/j.pmedr.2018.11.011
3. Amrock SM, Zakhar J, Zhou S, Weitzman M. Perception of e-cigarette harm and its correlation with use among U.S. adolescents. *Nicotine & Tobacco Research*. 2015; 17(3): 330-336. doi:10.1093/ntr/ntu156
4. Anand V, McGinty KL, O'Brien K, Guenthner G, Hahn E, Martin CA. E-cigarette use and beliefs among urban public high school students in North Carolina. *The Journal of Adolescent Health: Official Publication of the Society for Adolescent Medicine*. 2015; 57(1): 46-51. doi:10.1016/j.jadohealth.2015.03.018
5. Antin TMJ, Hess C, Kaner E, Lipperman-Kreda S, Annechino R, Hunt G. Pathways of nicotine product use: A qualitative study of youth and young adults in California. *Nicotine & Tobacco Research.* 2019; 1-6. doi:10.1093/ntr/ntz028
6. Barrington-Trimis J, Berhane K, Unger JB et al. Psychosocial factors associated with adolescent electronic cigarette and cigarette use. *Pediatrics*. 2015; 136(2): 308-317. doi:10.1542/peds.2015-0639
7. Bernat D, Gasquet N, Wilson KO, Porter L, Choi K, Wilson KO. Electronic cigarette harm and benefit perceptions and use among youth. *American Journal of Preventive Medicine*. 2018; 55(3): 361-367. doi:10.1016/j.amepre.2018.04.043
8. Bold KW, Kong G, Cavallo DA, Camenga DR, Krishnan-Sarin S. Reasons for trying E-cigarettes and risk of continued use. *Pediatrics*; 2016:138(3): doi:10.1542/peds.2016-0895.
9. Evans-Polce R, Patrick ME, Lanza ST, Miech RA, O'Malley PM, Johnston LD. Reasons for vaping among U.S. 12th graders. *The Journal of Adolescent Health: Official Publication of the Society for Adolescent Medicine.* 2018; 62(4), 457-462. doi:10.1016/j.jadohealth.2017.10.009
10. Gorukanti A, Delucchi K, Ling P, Fisher-Travis R, Halpern-Felsher B.Adolescents' attitudes towards e-cigarette ingredients, safety, addictive properties, social norms, and regulation. *Preventive Medicine.* 2017; 94: 65-71. doi:10.1016/j.ypmed.2016.10.019
11. Ickes M, Hester JW, Wiggins AT, Rayens MK, Hahn EJ, Kavuluru R. Prevalence and reasons for JUUL use among college students*. Journal of American College Health*. 2019; 1-5. doi:10.1080/07448481.2019.1577867
12. Khoury M, Manlhiot C, Fan CS et al. Reported electronic cigarette use among adolescents in the Niagara region of Ontario. C*MAJ: Canadian Medical Association Journal = Journal De L'Association Medicale Canadienne*. 2016; 188(11): 794-800. doi:10.1503/cmaj.151169
13. Kong G, Morean ME, Cavallo DA, Camenga DR, Krishnan-Sarin, S. Reasons for electronic cigarette experimentation and discontinuation among adolescents and young adults. *Nicotine & Tobacco Research: Official Journal of the Society for Research on Nicotine and Tobacco*. 2015; 17(7): 847-854. doi:10.1093/ntr/ntu257
14. Leavens ELS, Stevens EM, Brett EI, Leffingwell TR, Wagener TL. JUUL in school: JUUL electronic cigarette use patterns, reasons for use, and social normative perceptions among college student ever users. *Addictive Behaviors*. 2019; 99: 106047-106047. doi:10.1016/j.addbeh.2019.106047
15. Lee YO, Pepper JK, MacMonegle AJ, Nonnemaker JM, Duke JC, Porter L. Examining youth dual and poly-tobacco use with e-cigarettes. Internation*al Journal of Environmental Research and Public Health.* 2018; 15(4): 699-711 doi:10.3390/ijerph15040699
16. Montreuil A, MacDonald M, Asbridge M, et al. Prevalence and correlates of electronic cigarette use among Canadian students: Cross-sectional findings from the 2014/15 Canadian student tobacco, alcohol and drugs survey. *CMAJ Open*. 2017; 5(2): E460-E467. doi:10.9778/cmajo.20160167
17. Park E, Kwon M, Gaughan MR, Livingston JA, & Chang, Y. (2019). Listening to adolescents: Their perceptions and information sources about e-cigarettes. Journal of Pediatric Nursing, 48, 82-91. doi:10.1016/j.pedn.2019.07.010
18. Patrick ME, Miech RA, Carlier C, O'Malley PM, Johnston LD, Schulenberg JE. Self-reported reasons for vaping among 8th, 10th, and 12th graders in the US: Nationally-representative results. *Drug and Alcohol Dependence.* 2016; 165: 275-278. doi:10.1016/j.drugalcdep.2016.05.01
19. Tsai J, Walton K, Coleman BN et al. Reasons for electronic cigarette use among middle and high school students - National Youth Tobacco Survey, United States, 2016. *Morbidity and Mortality Weekly Report.* 2018; 67(6): 196-s200. doi:10.15585/mmwr.mm6706a5
20. Vogel EA, Ramo DE, Rubinstein ML. Prevalence and correlates of adolescents' e-cigarette use frequency and dependence. *Drug and Alcohol Dependence*. 2018; 188: 109-112. doi:10.1016/j.drugalcdep.2018.03.051
